# Mental Health and Tuberculosis—Holding Our Breath in Isolation

**DOI:** 10.3201/eid3003.AC3003

**Published:** 2024-03

**Authors:** Rena Fukunaga, Patrick K. Moonan

**Affiliations:** Centers for Disease Control and Prevention, Atlanta, Georgia, USA

**Keywords:** tuberculosis, TB, mental health, art and medicine, emerging infectious diseases, depression, anxiety, addiction, about the cover, art science connection, public health

**Figure:**
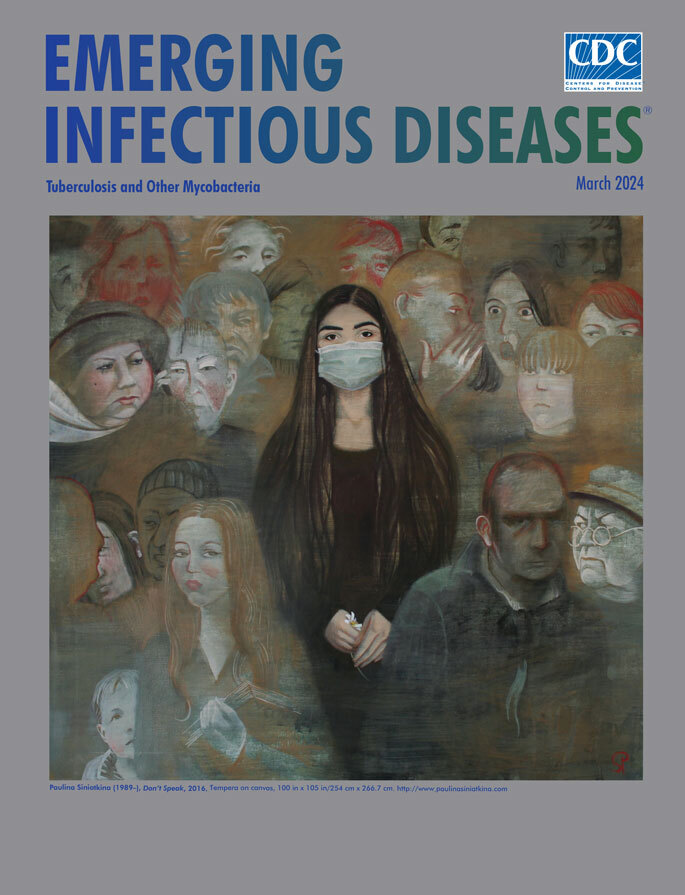
**Paulina Siniatkina (1989–), *Don’t speak!*** Tempera on canvas (2016), 37.4 in × 41.3 in/100 cm × 105 cm. http://www.paulinasiniatkina.com

Paulina Siniatkina, an artist and activist, is a survivor of tuberculosis (TB). In 2015, in a TB hospital on the outskirts of Moscow, the treating physician advised her to never talk about her TB diagnosis to anyone—further reinforcing the longstanding stigma associated with the disease. During her 7 months of treatment in isolation, Paulina experienced firsthand the suffering and loss associated with TB and turned to art to express her emotions and frustrations. She now uses her artistic talent and personal experience to advocate in the global fight against TB, and her work has drawn international recognition by the American Medical Association and World Health Organization. This month’s cover image, *Don’t speak!*, by Ms. Siniatkina, exemplifies the poignant psychology associated with TB. At the center, a young woman with sullen eyes draws your attention with her gaze, using a silent expression of longing to tell her story from behind the mask. Her unspoken feelings of hopelessness and depression appear to be subtly calmed by her nervous plucking of white petals from the single daisy protected by her hand, as the surrounding community dissolves into the background with looks of fear and judgement.

TB remains one of the leading causes of death by an infectious disease agent. Each year, more than 10 million people suffer from TB, and 1.5 million die as a result. Although curable, TB is a chronic multisystem infectious disease with well-documented, and often life-changing, disability and reduced quality of life. Treatment requires a multidrug, multimonth course of antibiotics; drug-resistant forms of TB extend the duration of treatment and in many communities require the patient to spend months in hospital or respiratory isolation. Not surprisingly, an estimated 40%–70% of persons treated for TB experience clinical anxiety or depression.

Beyond stigma and social isolation, mental illness persists as a silent driver of the global TB epidemic. Mental illness is associated with acquired drug resistance, TB transmission, disease recurrence, and TB-related death. Mental illness and TB are often exacerbated by homelessness and HIV co-infection. Integrated services for persons with TB and concurrent psychiatric conditions such as addiction, anxiety, or depression are now considered an essential component of global TB elimination efforts. However, in many countries with high burdens of TB, access to psychiatric services, including routine mental health screening and treatments, remain extremely limited.

Each year on March 24, we commemorate World TB Day in honor of the day Robert Koch announced to the Berlin Physiologic Society that he had discovered the cause of tuberculosis. World TB Day is a time to remember the millions of persons who suffer from TB, often in silence. It is also a time to break the silence, raise greater awareness, take specific actions to reduce the impact of mental health on our ambitions for global TB elimination, and not hold our breath in isolation.
